# dictyExpress: a *Dictyostelium discoideum *gene expression database with an explorative data analysis web-based interface

**DOI:** 10.1186/1471-2105-10-265

**Published:** 2009-08-25

**Authors:** Gregor Rot, Anup Parikh, Tomaz Curk, Adam Kuspa, Gad Shaulsky, Blaz Zupan

**Affiliations:** 1 Faculty of Computer and Information Science, University of Ljubljana, SI-1000 Ljubljana, Slovenia; 2 Graduate Program in Structural Computational Biology and Molecular Biophysics, Baylor College of Medicine, Houston, TX 77030, USA; 3 Department of Molecular and Human Genetics, Baylor College of Medicine, Houston, TX 77030, USA; 4 Department of Biochemistry and Molecular Biology, Baylor College of Medicine, Houston, TX 77030, USA

## Abstract

**Background:**

Bioinformatics often leverages on recent advancements in computer science to support biologists in their scientific discovery process. Such efforts include the development of easy-to-use web interfaces to biomedical databases. Recent advancements in interactive web technologies require us to rethink the standard submit-and-wait paradigm, and craft bioinformatics web applications that share analytical and interactive power with their desktop relatives, while retaining simplicity and availability.

**Results:**

We have developed dictyExpress, a web application that features a graphical, highly interactive explorative interface to our database that consists of more than 1000 *Dictyostelium discoideum *gene expression experiments. In dictyExpress, the user can select experiments and genes, perform gene clustering, view gene expression profiles across time, view gene co-expression networks, perform analyses of Gene Ontology term enrichment, and simultaneously display expression profiles for a selected gene in various experiments. Most importantly, these tasks are achieved through web applications whose components are seamlessly interlinked and immediately respond to events triggered by the user, thus providing a powerful explorative data analysis environment.

**Conclusion:**

dictyExpress is a precursor for a new generation of web-based bioinformatics applications with simple but powerful interactive interfaces that resemble that of the modern desktop. While dictyExpress serves mainly the *Dictyostelium *research community, it is relatively easy to adapt it to other datasets. We propose that the design ideas behind dictyExpress will influence the development of similar applications for other model organisms.

## Background

Public databases of results from high-throughput experiments are abundant and extremely useful, but most biologists lack the training in computer programming to effectively explore and interact with the data. A solution to this problem is afforded by recent developments in information technology, which facilitate the development of web-based systems that support interaction and explorative data analysis. These systems require only basic web-surfing skills and modest computer power, but may deliver powerful data analysis capabilities to the biologist's fingertips. The major advantages of these systems over first-generation web applications is that they provide the look and feel of a desktop application within a web browser window, with intuitive visualization and the availability of helpful hints through techniques such as term-completion and tagging. Instead of the infamous "Submit" button and the corresponding switch from one web page to another, modern systems employ an interactive, single-screen interface, which adapts to the user's data and actions. Such interfaces are used in many popular applications, such as e-mail browsers, spreadsheets, words processors, and social networking websites, but the field of bioinformatics has yet to adopt these new technologies.

In this paper, we report on the development of a gene expression database and its corresponding web application, dictyExpress , that we consider to be a pioneering attempt at this change. This application is designed around a repository of gene expression data from microarray experiments in the social amoeba *Dictyostelium discoideum*. *Dictyostelium *is a popular organism in which individual cells aggregate upon starvation and differentiate into fruiting bodies that consist of two major cell types – spores and stalks. This organism is a convenient model system for the study of cell motility, chemotaxis, development, social behaviour and more, and the availability of global gene expression profiles greatly facilitates these studies. Until now, gene expression profiles of this popular system have been deposited in public databases and some of them are available as static graphs on the organism's central web site, dictyBase . These graphs depict individual gene expression profiles as they change during development of the wild type strain, but they do not allow curious biologists to explore gene expression in other strains, or interact with the data in any other way.

Dealing with time is central in *Dictyostelium *development. dictyExpress was developed to specifically address time, and to provide optimized procedures for retrieval, visualization and interactive analysis of time-series data. The result of this effort is a comprehensive, electronically-accessible database of all the *Dictyostelium *expression data published by the Functional Genomics Project at Baylor College of Medicine, featuring a web-based application that can query the database and perform sophisticated data mining tasks. The web interface can be easily expanded to include additional tools and adapted to the analysis of other public databases. In this paper, we provide the overall description of both the database and the website components of dictyExpress. We then focus on the web-based exploratory environment, which we regard as the major original contribution of our work.

## Implementation

**Figure 1:**
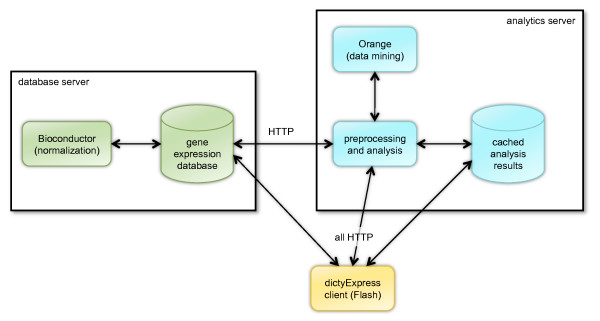
**The architecture of dictyExpress**. The three major components of dictyExpress are a database server, and analytics server, and a client implementing graphical user interface that communicates with both servers using HTTP queries.

dictyExpress is composed of a MS SQL database, software for data management and retrieval, data analysis software and a client web-based application (Figure 1). The data management and retrieval part is written within MS SQL, and it interfaces with the Bioconductor suite [1] for data normalization and pre-processing. The system provides data to external clients through a programmatic, HTTP-based interface. The client web application, developed in FLASH within the FLEX open source development framework , offers a number of data analysis procedures, mainly relying on an analytics server that either performs the analysis on the fly (e.g., clustering, or network construction), or fetches stored results of previous analyses (e.g., gene similarity scoring). The dictyExpress database server is freely accessible. In contrast, the dictyExpress analytics server, which also implements an HTTP-based programmatic interface, is available only to dictyExpress client-specific requests. The analytics server is powered by the Orange data mining suite [2,3] to process profile similarity scoring, clustering and dendrogram optimization, and Gene Ontology term enrichment analysis.

A critical issue with dictyExpress is the performance of the analytics server, which carries all the computational load of the application and is a potential bottleneck in the system. We have developed a dedicated web application to estimate the response time under different loads of the server, and are monitoring its performance daily. An expansion to a server farm is planned if the current, multi-core server will become insufficient.

## Results and discussion

**Figure 2:**
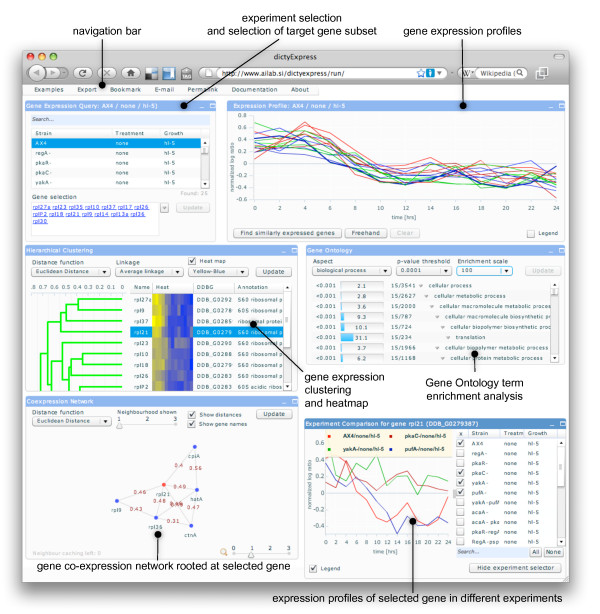
**Components of the dictyExpress user interface**. The five major components of the dictyExpress graphical interface include experiment and gene selection, display of expression profiles, gene ontology term enrichment analysis, clustering analysis, gene co-expression network, and a display of expression profiles of a selected (target) gene in various experiments. The example shows a group of genes that encode ribosomal proteins. These genes are sharply down-regulated upon starvation of the organism.

dictyExpress provides access to over 1057 expression array experiments from the BCM Functional Genomics group. The web interface (Figure 2) includes components for data retrieval, selection of individual genes or groups of genes, graphic display of gene expression time courses, Gene Ontology term enrichment analysis, display of gene co-expression networks, hierarchical clustering, and expression profile visualization of a selected gene in different experiments. Most importantly, the components are "connected" such that a change in any one of the components (e.g., selection of a gene subset from the hierarchical clustering dendrogram) can propagate to other components and their associated visualizations. Visual interaction is also incorporated into data retrieval: a user can, for example, hand-draw an approximate gene expression profile and dictyExpress would find and display genes that match it.

### Content of the dictyExpress database

As of March 2009, the dictyExpress database included data from over 1057 expression array experiments collected from wild type and from 24 mutant strains that were grown in HL5 liquid suspension and developed on nitrocellulose filters [4-12]. All of the microarrays were printed with cDNA and genomic DNA fragments that were amplified by PCR. All the experiments were done by comparative hybridization where each sample (e.g., a particular time point in development) was compared with a common pool of RNA samples form various time points in *Dictyostelium *development. In most of the experiments, gene expression was observed at 13 different time points (2-hour intervals during the 24-hour developmental time course). A list of *Dictyostelium *strains, time points at which the data were recorded, and the number of replication measurements is provided on the application home page.

### User interface and analytical functions

Six different components make up the graphical user interface of the dictyExpress web application:

- *Gene Expression Query *is where the user selects the desired experiments and defines the genes of interest. Experiment filtering (strain name, treatment or growth conditions should match all of the entered search terms) and gene name completion are also supported. The names in the gene selection list are clickable and the links lead to the corresponding dictyBase gene home page.

- *Expression Profile *displays gene expression profiles (time-series) for the given experiment and the set of selected genes. Any profile can be selected form the visualization, and may be followed by a request to search for genes with similar expression profiles. The user may also hand-draw a hypothetical profile using the 'freehand' option and search for genes with a similar behaviour.

- *Hierarchical Clustering *considers a list of selected genes and displays a corresponding dendrogram with associated gene names, gene IDs and textual annotations. A heat map, representing the expression time-series, can also be visualized, thus providing a standard dendrogram-heat map visualization that researchers have become familiar with in recent years [13]. The dendrogram is clickable, allowing the user to select any branch and its corresponding genes.

- *Gene Ontology *displays the results of a term-enrichment analysis in which the selected genes serve as the query and all the genes from the experiment serve as the reference set. The display follows the visual design proposed by the enrichment analysis application GOAT [14], and provides information on the enrichment scores and associated *p*-values. Reports on each of the three Gene Ontology aspects (molecular function, biological process and cellular component) can be accessed separately.

- *Co-expression Network *considers a single reference gene and displays a neighbourhood network of the most similar genes. The application uses the same profile similarity measures as the gene clustering application. The networks are drawn so that the most distant gene in the network is up to 3 hops away from the target gene. Double-clicking on any gene makes it the reference component, and results in redrawing the network accordingly.

- *Experiment Comparison *displays expression profiles of a reference gene in several (selected) experiments. Double-clicking on any of the profiles makes the corresponding experiment the current one and updates the content of all the other components, while retaining the current selection of genes.

The web-based interface of dictyExpress also implements a set of supporting functions that are available through the applications menu bar (see Figure 2, upper left part of the snapshot). These include data export (of selected genes or the entire experiment), bookmarking of the current view (the experiment and the selected genes will be stored in a bookmark) or, alternatively, copying the permalink (web link to the application and current selection of experiment and genes) to the clipboard, sending an e-mail with an embedded link to the current view, and crafting a PDF report of the current view.

### Explorative data analysis

The central design principle used in crafting the dictyExpress web application was interactivity. Most of the visual presentations we implement are clickable: the user can select a gene subset by clicking on a branch of the dendrogram or select a term in the Gene Ontology annotation. A gene in the co-expression network can be selected by clicking on any of the networks nodes, by selecting the corresponding trajectory in the Expression Profile component, or by selecting a corresponding column in the hierarchical clustering dendrogram. Gene selections are communicated across the different components of the application, either upon manual request (pressing the "Update" button in the corresponding window would modify the set of selected genes in all the other components) or automatically (e.g., selection of a single gene in the dendrogram highlights the corresponding profile in the Expression Profiles and triggers the rendering of a co-expression network around this particular gene).

**Figure 3:**
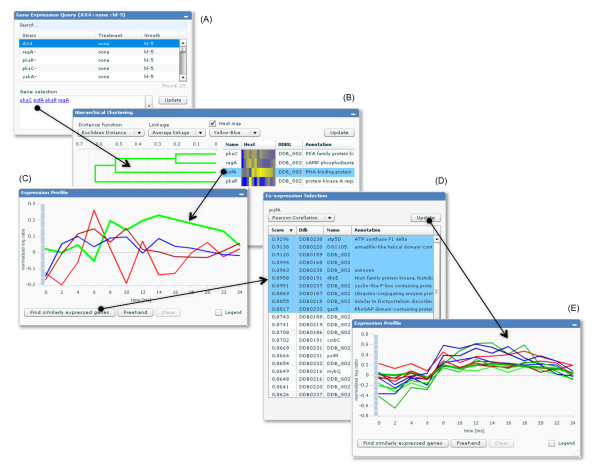
**An example of explorative data analysis in dictyExpress**. An example of transcriptome exploration steps enabled in dictyExpress. The user starts with a selection of an experiment and a set of genes (A). The user continues by selecting a target gene profile from the dendrogram (the *pufA *gene) (B). The application automatically highlights the corresponding trajectory in the profile visualization (C). The next step is a request to find and display of list of similarly expressed genes (D). The user can select a subset of the most similar genes, making this list the active set of genes, with a subsequent update of all the dictyExpress components, including the one showing the gene expression profiles (E).

Another useful feature implemented in dictyExpress is an explicit search for similarly expressed genes (Figure 3). The user selects a target gene in the Expression Profile or in the Hierarchical Clustering components and the search is invoked by pressing the "Find similarly expressed genes" button.

All of the features listed above contribute to the implementation of explorative data analysis, where the visual presentation of the data guides the user on which genes to focus next and what gene expression profiles to consider. This interactivity sets dictyExpress apart from other current web-based gene expression analysis applications.

### Electronic access to the dictyExpress database

The data in the dictyExpress repository are available through a programmatic (HTTP) data access. The user can query for raw microarray data, normalized data, and time series data, obtain various mapping schemas, such as the one between microarray spot ID and gene ID, and access various annotations of the experiments. The following links are a few examples of HTTP queries in dictyExpress:

-  returns a mapping from microarray spots to gene names and IDs

-  lists all the biological samples in the data base

-  returns the time series expression data for six genes (given are their repository IDs) for an experiment done with the *mek1*^- ^knockout mutant where no treatment was applied and where the growth conditions are not specified.

The detailed documentation on the syntax of HTTP dictyExpress queries, along with more examples that illustrate their use, is available on-line at .

## Conclusion

dictyExpress provides a public database of gene expression data for a popular model organism and an original web-based application for data exploration. The prototype of the system has been launched in September 2008. As of April 2009, dictyBase includes direct links to dictyExpress from the gene home pages. Following these links, dictyExpress displays the wild-type expression profile for a selected gene, and supports the user for further exploration. The users are able to test the expression of their gene of interest in the wild type and in the mutant strains, and use the application for exploring the *Dictyostelium *transcriptome, shuttling back and forth to dictyBase in order to explore the annotations of interesting genes.

dictyExpress has an enormous value to the entire community. Currently, it allows researchers to explore a large database of expression arrays without having knowledge in data mining or access to powerful computers and expensive software. Eventually, we anticipate that other research communities will adopt this approach so that public databases of gene expression arrays would become readily available to the public.

## Availability and requirements

dictyExpress can be accessed at . The software is platform-independent and runs in all major browsers with a Flash player plug-in. It has been tested within the Firefox, Internet Explorer and Safari web browsers.

## Methods

### Microarray normalization and pre-processing

The pre-processing pipeline incorporates the LIMMA package [15] in R/BioConductor [1] for quality control and normalization. Within chip normalization is performed using the 'printtip loess' normalization function in LIMMA. Along with normalized intensities two statistics are calculated for each chip: chip quality, and median signal intensity. Chip quality is characterized by correlation between on-chip replication, and can be used to filter for high quality chips. Median signal intensities are stored for efficient scaling for between chip normalization.

### Gene expression profile similarity scoring

Gene expression profiles in dictyExpress are time sequences mainly consisting of 13 developmental time points that were analyzed with a custom cDNA array (Van Driessche et al. 2002). Several analysis components, including the one for clustering, gene co-expression network construction and search for similar expression profiles rely on the scoring of a pair-wise profile distance. We use three alternative standard similarity measures: Euclidian distance (squared sum of squared per-point distances), Pearson's correlation (standard Pearson product-moment correlation coefficient) or Manhattan distance (squared sum of absolute per-point distances).

### Gene expression clustering

Gene expression profiles are clustered using agglomerative hierarchical clustering [16], where the user can choose between average, single, or complete linkage. The layout of the dendrogram is optimized to minimize the gene expression profile distances between neighbouring leaves [17].

### GO term enrichment analysis

The hypergeometric distribution is used to compute the enrichment of Gene Ontology terms [18] for the selected subset of genes. All the other genes in the experiment constitute the reference set. For the display of the results we have borrowed the visualization from GOAT [14], which shows ontology branches of enriched terms together with the corresponding *p*-values and enrichment scores.

### Gene co-expression network

Co-expression networks are developed around a user-selected target gene and its neighbours with similar expression profiles. For reasons of obtaining reasonable response times, we use the following heuristics to define which genes to consider in the network. Network genes are gathered starting from the target gene, including its five most similar neighbours and the procedure is iteratively repeated on the newly added genes up to three times (according to a user-defined parameter). Only edges that link the most similar genes are shown in the visualization, and they are selected such that the average connectivity of the genes in the network is 5.0. The networks are developed on the analytics server from pre-processed data that includes a list of 20 closest neighbours for each similarity measure and each gene. This particular network construction algorithm is speed-efficient and heuristically constructs networks that are close to those we would obtain through extensive search and computation of the entire similarity matrix.

## Authors' contributions

BZ and GS conceived the project and the initial design. AP developed the dictyExpress database server and its HTTP interface. GR implemented the web-based interface and designed the corresponding web pages. Server-based data analysis scripts were implemented by TC, BZ and GR. GS and AK provided the test data and performed user testing. All the authors drafted the manuscript and read and approved its final version.
